# The hospital exemption pathway for the approval of advanced therapy medicinal products: an underused opportunity? The case of the CAR-T ARI-0001

**DOI:** 10.1038/s41409-021-01463-y

**Published:** 2022-01-19

**Authors:** Esteve Trias, Manel Juan, Alvaro Urbano-Ispizua, Gonzalo Calvo

**Affiliations:** 1 Advanced Therapies Unit, Hospital Clínic Barcelona, Barcelona, Spain; 2 Immunotherapy–Immunology Department, Hospital Clínic Barcelona, Barcelona, Spain; 3 Hemato-Oncology Institute, IDIBAPS, Hospital Clínic Barcelona, Barcelona, Spain; 4 Clinical Pharmacology Department, Division of Medicines, Hospital Clínic Barcelona, Barcelona, Spain

**Keywords:** Immunotherapy, Gene therapy, Haematological cancer

## Abstract

In February 2021, the ‘Advanced Therapy Medicinal Product’ (ATMP) ARI-0001 (CART19-BE-01), developed at Hospital Clínic de Barcelona (Spain), received authorization from the Spanish Agency of Medicines and Medical Devices (AEMPS) under the ‘hospital exemption’ (HE) approval pathway for the treatment of patients aged >25 years with relapsed/refractory (RR) acute lymphoblastic leukemia (ALL). The HE pathway foreseen by the European Regulation establishing the legal framework for ATMPs intended to be placed on the market in the EU, allows access to ATMPs prepared on a non-routine basis, according to quality standards, like a custom-made product for an individual patient. Its use is limited to the same Member State where it was developed, in a hospital under the responsibility of a medical practitioner. HE-ATMPs must comply with national traceability and pharmacovigilance requirements and specific quality standards. HE offers an opportunity to develop ATMPs in close contact with clinical practice, with the quality and rapid access needed by patients and at a lower cost compared to regular market authorization. However, many barriers need to be overcome. Here we discuss relevant aspects of the development and authorization of ARI-0001 in the context of the heterogeneous frame of the European Regulation implementation across the Member States.

In February 2021, the ‘Advanced Therapy Medicinal Product’ (ATMP) ARI-0001 (CART19-BE-01, an anti-CD19 CAR [Chimeric Antigen Receptor or CAR19] against CD19+ B-cell malignancies) received authorisation from the Spanish Agency of Medicines and Medical Devices (AEMPS) under ‘hospital exemption’ (HE) for the treatment of adult patients (>25 years old) with relapsed/refractory (R/R) acute lymphoblastic leukaemia (ALL) [1]. This is an important landmark given that it is the first CAR to have been developed from bench to bedside in the European Union (EU)—more specifically in Barcelona, Spain—and the first to receive the authorisation of a governmental drug agency outside the centralised marketing authorisation (MA) pathway (i.e. under non-industrial conditions but similar quality, safety and efficacy standards) foreseen by the European ATMP Regulation [2]. Moreover, this authorisation takes place in the scenario of a rare, life-threatening disease with a dismal prognosis in the R/R situation, where there are few, if any, remaining therapeutic alternatives. Thus, the availability of ARI-0001 provides a model of ATMP development that is close to clinical practice in many respects, exemplifying thus “how initiatives from Academia and Pharmaceutical companies can be complementary, working in the best interest of patients” [3]. These relevant aspects are discussed in depth below.

ALL is a rare disease with an estimated overall annual incidence in Europe of 1.28 per 100 000 adult individuals [4]. B-cell is the cell type of origin in 85% of cases [5] (B-ALL), the other cell type being T-cell (T-ALL). Unlike in children with B-ALL, the prognosis of adults with B-ALL is poor, with cure rates of barely 40% [6]. Prognosis is even worse for R/R patients, with complete remission (CR) rates of 20–40%, median overall survival (OS) of 6 months and cure rates <10% despite intensive salvage chemotherapy and haematopoietic stem cell transplantation [6]. In the past few years, antibody-targeted therapies directed against cell surface antigens CD20, CD19 and CD22 have been a breakthrough. Among these, monoclonal antibodies such as blinatumomab (CD19) and inotuzumab (CD22) have increased CR and extended OS in these patients in the salvage setting, although duration of response was short (median of 7.3 and 4.6 months, respectively) [7, 8] and impact on OS was limited, among other limitations [9]. Several trials are investigating new antibody-targeted therapies as well as optimal combinations and sequences [6, 9]. In the meantime, R/R B-ALL continues to be a major clinical challenge in adults [4] for whom further therapeutic options impacting quantity and quality of life are urgently needed.

CAR-T therapies have revolutionised the management of both paediatric and adult R/R B-cell lymphoproliferative disorders [9]. Among the current generations of CAR-Ts [6, 10], four autologous CAR19 T-cell products—tisagenlecleucel (Kymriah^®^, Novartis), axicabtagene ciloleucel (Yescarta^®^, Gilead), brexucabtagene autoleucel (Tecartus^®^, Gilead) and lisocabtagene maraleucel (Breyanzi^®^, Bristol Myers Squibb)—have been approved by the US Food and Drugs Administration (FDA) and the European Medicines Agency (EMA). More recently (2021), idecabtagene vicleucel (Abecma^®^, Bristol Myers Squibb) has received FDA approval and is currently awaiting EMA approval. The first approval of CAR-T cells as a tool to fight cancer was the result of near 30 years of robust research [11], since the first report of chimeric combination of receptors and antibodies was published in 1989 [12]. CAR19 T-cell therapy has been rapidly adopted for the treatment of several B-cell malignancies [13]. It is important to remark that this is an autologous therapy, customised for each individual patient. Although the preclinical data and even the first clinical data were obtained in an academic environment, MA-approved CAR19 T-cell products must be ordered from the pharmaceutical company that holds the licence and their production is subject to manufacturing capability according to the demand. Time is of the essence in this setting.

ARI-0001 was developed at Hospital Clínic de Barcelona, Spain. It is a second-generation CAR, built upon a new construct based on A3B1 (a monoclonal antibody developed by the Hospital Clínic de Barcelona more than 30 years ago) combined with CD8:41BB:CD3z regions; the lab work for this CAR-T began in 2013 [14, 15]. After obtaining positive results in preclinical experiments and demonstrating robust and reproducible lentivirus and ARI-0001 cell production, its safety and efficacy were assessed in a study conducted together with the paediatric hospital Sant Joan de Déu (Barcelona). In this study participated 47 patients with R/R lymphoproliferative B disorders, with a median age of 47.5 years (range 3–67), of which ALL accounted for 80% of patients. The overall safety and efficacy outcomes achieved were similar to those of other academic and commercial products [3]. ARI-0001 is currently being further investigated in a Phase 2 study in adult patients with R/R CD19+ ALL (NCT04778579). Figure 1 shows the major deliverables in the whole ARI-0001 development and authorisation processes, as well as the current state of the project.

**Fig. 1:**
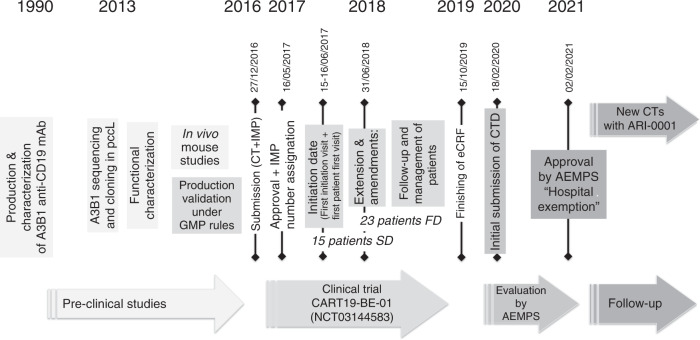
Main deliverables during the ARI-0001 development and authorisation processes and current state. Under the time line from 1990 to 2021, each square with vertical text show aspects related with the development; the vertical text with a specific date express more relevant milestones in the process; horizontal text inside arrows express main steps of this process. The number of patients in horizontal are located in the general period of the timeline.

ARI-0001 was authorised under the HE approval pathway foreseen by European Regulation [EC] No 1394/2007 on ATMPs and amending Directive 2001/83/EC and Regulation [EC] No 726/2004, which establish the legal framework for ATMPs that are intended to be placed on the market in the EU [2]. Conversely to a standard centralised MA, which ensures broad access to all Member States, HE allows the access to ATMPs “prepared on a non-routine basis, according to specific quality standards, as a custom-made product for an individual patient” [2]. Their use is limited to the same Member State where it was developed, in a hospital under the exclusive professional responsibility of a medical practitioner [2]. Notwithstanding the different authorisation pathways, HE-ATMPs must be authorised by the competent authority of the Member State and must comply with national traceability and pharmacovigilance requirements and specific quality standards [2]. ARI-0001’s manufacturing approval following Good Manufacturing Practices (GMP) in 2017 preceded the authorisation in 2021 to treat R/R ALL adult patients in Hospital Clínic de Barcelona under HE. The authorisation of ARI-0001 under HE follows that of NC1—autologous expanded mesenchymal stromal cells and autologous plasma developed by Hospital Puerta de Hierro, Madrid—which was authorised in January 2019 for patients with chronic traumatic spinal cord sequelae [16]. They are the only two HE-ATMPs authorised in Spain [17].

HE undoubtedly offers an opportunity to develop ATMPs in close contact with clinical practice and with the rapid access needed by patients. However, many barriers are yet to be overcome to take full advantage of it. The first is the need to harmonise HE rules across Member States [18, 19], whose current heterogeneity as a result of differences in interpretation and of implementation of the European legislation hamper transnational collaboration in HE-ATMPs [19, 20]. This heterogeneity is mainly the result of differences in the interpretation of the criteria that define HE [20–23], the lack of clarity in relevant aspects of national legislation on ATMPs [19, 20, 23] and the flexibility provided by ATMP-specific GMP guidelines [24]. Both academia and industry are demanding harmonisation in HE rules [18], although the present discrepancies allow us to test pros and cons of these differences in a real situation, providing data about the best decisions for this harmonisation. Diseases with largely unmet medical needs deserve solutions that provide not only quality and safety but also evidence of efficacy [19].

ARI-0001 was developed and brought to the patient bedside following the Spanish legislation on ATMPs (RD 477/2014), which not only complies with the strict requirements of the European ATMP legislation on quality and safety but also requires the provision of clinical data on efficacy and safety prior to HE authorisation [25]. These requirements are not uniformly applied across the EU [23]. A ‘conditional licence’ is granted for three years, which is subject to a follow-up annual report, fulfilment of specific regulatory obligations and a re-evaluation of the data for a further 5-year renewal [25]. These requirements are to increase the evidence on efficacy and safety in the absence of evidence stemming from Phase 3 studies. This is in opposition to the ‘conditional authorisation’ given to centrally approved products for an indefinite period of time, only subjected to the provision of additional data. However, the procedure foreseen in the Spanish legislation is not agile enough to allow the development of new CAR-T therapies with the speed needed by patients with potentially life-threatening diseases for whom treatment alternatives are lacking. This is primarily because any change (either minimal or substantial) in the product is seen as a ‘new product’ in the eyes of the legislation, which means starting the development process (including generation of evidence) from scratch, with limited possibilities of bridging data between dossiers. This situation is a significant barrier to product improvement. The autologous origin of the product should help to make this aspect more flexible to help achieve faster improvement, especially when the proposed improvements do not involve substantial changes of the product.

ATMP manufacturing under HE requires a large amount of human, logistic and financial resources, which, together with regulatory challenges, commonly poses a great barrier for public facilities, but also for private investors (e.g. small and medium enterprises). ARI-0001 was possible thanks to the work and expertise of a multidisciplinary team that participated in the successive steps of this process, from the development and reshaping of the product, its application and the conduct of clinical trials demonstrating its safety and efficacy to pharmacovigilance activities (Fig. 2); the availability of logistic resources (clean room with CAR-T manufacturing facilities) is also a key pillar. Both reflect the commitment of Spain, and specifically Catalonia, to R&D&I in general. ARI-0001 was financially possible thanks to the support of public authorities and civil society, including the ‘ARI Project’, under which donations were made by nearly 1500 individuals, 23 associations and foundations, and 56 companies.

**Fig. 2:**
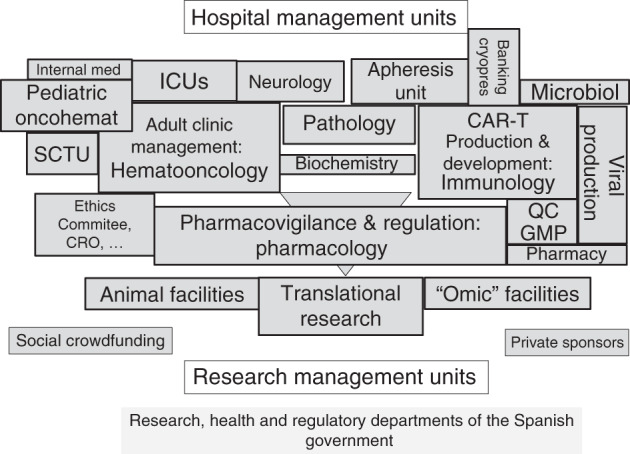
The different font sizes indicate the relative participation in the project. CRO contract research organization, QC GMP quality control according to good manufacturing practices, ICU intensive care unit, SCTU stem cell transplantation unit.

Another significant limitation is how to make HE-ATMPs accessible to patients from hospitals other than the hospital that developed the product [26]. This limitation is even more critical in Spain, where national legislation only grants the licence to the hospital that developed the product [22]. Technology transfer and know-how are essential to facilitate access to HE-ATMPs in other hospitals within or outside the Member State and to help reduce manufacturing costs [22]. The European ‘Common representation of Substances of Human Origin’s’ (CoRe SoHo) [27], whose goal is “to provide representative technical expertise to the European decision-making organisations in the field of SoHo”, plays an important role here by encouraging transparency and exchange among hospitals. This need has been foreseen by the AEMPS with the designation of the HE-ATMP developer as the ‘Reference hospital’ [25]. By participating in the mentioned multicentre Phase 2 clinical trial for adult R/R ALL patients with the reference hospital, other Spanish hospitals will accumulate efficacy and safety evidence with a view to applying to the AEMPS for authorisation of HE-ATMPs [25].

The availability of ATMPs is very much influenced by the huge costs associated with bringing them to the patient’s bedside, which makes the return on investment challenging for pharmaceutical companies, especially when considering the small size of populations addressed [28]. Not surprisingly, of the 12 ATMPs that have been granted MA by the EMA since 2009, four were withdrawn from the market during 2018 for ‘commercial reasons’, in some cases due to lack of reimbursement [29]. Although ATMPs development following the HE pathway does not pursue saving costs but rather to provide a solution for patients for whom no treatment alternatives are available, it is worth noting the reduced cost associated to developing and manufacturing HE-ATMPs. Despite this might benefit public and private payers, it should always be taken into account that HE-ATMPs are available to a very limited number of patients (i.e. those of the developer hospital). The price of ARI-001 under the HE-ATMPs pathway is one-third of the commercial CAR-Ts available in Spain. Safe, effective and equitable access to CAR-T therapy has been granted since 2019 by the Spanish National Health System (NHS), which, given the high cost of these therapies, has made reimbursement conditional on the therapeutic value provided—i.e. on real benefits in clinical practice—[30]. Conversely, NC1 has been reimbursed by the Spanish NHS since October 2019 [31]. ARI-0001 aims to be the ‘first publicly-owned CAR-T’.

To summarise, the HE pathway provides the necessary speed to treat patients and allows treatment and dosage to be customised according to patients’ specific characteristics and needs. By reducing manufacturing costs, the therapies are more affordable for public and private payers. However, some limitations are yet to be overcome. A homogeneous HE pathway across the EU could ensure common high quality, safety, and efficacy standards, although this harmonisation should aim to minimise the level of limitations, seeking the improvement of developments that could help patients. Although the requirements of the Spanish legislation on ATMPs are very strict, in accordance with the EU Regulation, they have allowed the development of a CAR-T cell therapy, warranting the standards of safety and efficacy required of commercial CAR-Ts. As for conditioned approvals applied for by current commercial CAR-Ts, outcomes for these academic CAR-Ts should be evaluated continuously, gaining evidence. Investing in human and logistic resources is essential for success, and fostering public-private financial cooperation is needed to undertake these complex projects. In any case, confidence in all these products is critical for this cooperation. Transmission of information and know-how is essential to facilitate access to HE-ATMPs for other hospitals. The Spanish legislation foresees the figure of the ‘Reference hospital’ to gain access in the Spanish setting, although other figures are needed at the European level. It is time to reflect on how the future of these proposals should be in order to help develop better treatments for our patients.

## References

[1] ARI-0001 Summary of Product Characteristics (Spanish). https://www.aemps.gob.es/investigacionClinica/terapiasAvanzadas/docs/ARI-0001_ficha-tecnica.pdf?x74012. Accessed May 2021.

[2] Regulation (EC) No 1394/2007 of the European Parliament and of the Council of 13 November 2007 on advanced therapy medicinal products and amending Directive 2001/83/EC and Regulation (EC) No 726/200425th of February. https://eur-lex.europa.eu/LexUriServ/LexUriServ.do?uri=OJ:L:2007:324:0121:0137:en:PDF. Accessed May 2021.

[3] Ortiz-Maldonado V, Rives S, Castella M, Alonso-Saladrigues A, Benitez-Ribas D, Caballero-Banos M (2021). CART19-BE-01: a multicenter trial of ARI-0001 cell therapy in patients with CD19(+) relapsed/refractory malignancies. Mol Ther.

[4] Hoelzer D, Bassan R, Dombret H, Fielding A, Ribera JM, Buske C (2016). Acute lymphoblastic leukaemia in adult patients: ESMO Clinical Practice Guidelines for diagnosis, treatment and follow-up. Ann Oncol.

[5] DeAngelo DJ, Jabbour E, Advani A (2020). Recent advances in managing acute lymphoblastic leukemia. Am Soc Clin Oncol Educ Book.

[6] Samra B, Jabbour E, Ravandi F, Kantarjian H, Short NJ (2020). Evolving therapy of adult acute lymphoblastic leukemia: state-of-the-art treatment and future directions. J Hematol Oncol.

[7] Kantarjian H, Stein A, Gokbuget N, Fielding AK, Schuh AC, Ribera JM (2017). Blinatumomab versus chemotherapy for advanced acute lymphoblastic leukemia. N Engl J Med.

[8] Kantarjian HM, DeAngelo DJ, Stelljes M, Martinelli G, Liedtke M, Stock W (2016). Inotuzumab ozogamicin versus standard therapy for acute lymphoblastic leukemia. N Engl J Med.

[9] Paul S, Rausch CR, Nasnas PE, Kantarjian H, Jabbour EJ (2019). Treatment of relapsed/refractory acute lymphoblastic leukemia. Clin Adv Hematol Oncol.

[10] Zhao J, Song Y, Liu D (2019). Clinical trials of dual-target CAR T cells, donor-derived CAR T cells, and universal CAR T cells for acute lymphoid leukemia. J Hematol Oncol.

[11] Wrona E, Potemski P (2019). A novel immunotherapy—the history of CAR T-cell therapy. Oncol Clin Pract.

[12] Gross G, Waks T, Eshhar Z (1989). Expression of immunoglobulin-T-cell receptor chimeric molecules as functional receptors with antibody-type specificity. Proc Natl Acad Sci USA.

[13] June CH, Sadelain M (2018). Chimeric antigen receptor therapy. N Engl J Med.

[14] Castella M, Boronat A, Martin-Ibanez R, Rodriguez V, Sune G, Caballero M (2019). Development of a novel Anti-CD19 chimeric antigen receptor: a paradigm for an affordable CAR T cell production at academic institutions. Mol Ther Methods Clin Dev.

[15] Castella M, Caballero-Banos M, Ortiz-Maldonado V, Gonzalez-Navarro EA, Sune G, Antonana-Vidosola A (2020). Point-of-care CAR T-cell production (ARI-0001) using a closed semi-automatic bioreactor: experience from an academic phase I clinical trial. Front Immunol.

[16] NC1 Summary of Product Characteristics (Spanish). https://www.aemps.gob.es/investigacionClinica/terapiasAvanzadas/docs/NC1_ficha-tecnica.pdf?x74012. Accessed May 2021.

[17] Agencia Española del Medicamento y Productos Sanitarios (AEMPS). Authorized ATMPS. https://www.aemps.gob.es/medicamentos-de-uso-humano/terapias-avanzadas/autorizaciones-de-uso-de-medicamentos-de-terapia-avanzada/. Accessed May 2021.

[18] Cuende N, Boniface C, Bravery C, Forte M, Giordano R, Hildebrandt M (2014). The puzzling situation of hospital exemption for advanced therapy medicinal products in Europe and stakeholders’ concerns. Cytotherapy..

[19] Alliance for Regenerative Medicine. Recommendations for the use of Hospital Exemption 2020. Available from: http://alliancerm.org/wp-content/uploads/2020/10/ARM-position-on-HE-final-Oct-2020.pdf. Accessed May 2021.

[20] Van Wilder P (2012). Advanced therapy medicinal products and exemptions to the regulation 1394/2007: how confident can we be? An exploratory analysis. Front Pharm.

[21] Ivaskiene T, Mauricas M, Ivaska J (2017). Hospital exemption for advanced therapy medicinal products: issue in application in the European Union member states. Curr Stem Cell Res Ther.

[22] Coppens DGM, Hoekman J, De Bruin ML, Slaper-Cortenbach ICM, Leufkens HGM, Meij P (2020). Advanced therapy medicinal product manufacturing under the hospital exemption and other exemption pathways in seven European Union countries. Cytotherapy..

[23] Hills A, Awigena-Cook J, Genenz K, Ostertag M, Butler S, Eggimann A-V (2020). An assessment of the hospital exemption landscape across European Member States: regulatory frameworks, use and impact. Cytotherapy..

[24] EudraLex. The Rules Governing Medicinal Products in the European Union Volume 4. Good Manufacturing Practice. Guidelines on Good Manufacturing Practice specific to Advanced Therapy Medicinal Products 2017. https://ec.europa.eu/health/sites/health/files/files/eudralex/vol-4/2017_11_22_guidelines_gmp_for_atmps.pdf. Accessed May 2021.

[25] Real Decreto 477/2014, de 13 de junio, por el que se regula la autorización de medicamentos de terapia avanzada de fabricación no industrial2014. https://www.aemps.gob.es/medicamentos-de-uso-humano/terapias-avanzadas/. Accessed May 2021.

[26] Report from the Commission to the European Parliament and the Council in accordance with Article 25 of Regulation (EC)2007 of the European Parliament and of the Council on advanced therapy medicinal products and amending Directive 2001/83/EC and Regulation (EC) No 726/2004. Available from: https://op.europa.eu/en/publication-detail/-/publication/2dc18b82-b6c8-11e3-86f9-01aa75ed71a1. Accessed May 2021.

[27] Common representation of Substances of Human Origin’s (CoRe SoHO). Available from: https://ec.europa.eu/transparencyregister/public/consultation/displaylobbyist.do?id=501652723968-72. Accessed May 2021.

[28] Seoane-Vazquez E, Shukla V, Rodriguez-Monguio R. Innovation and competition in advanced therapy medicinal products. EMBO Mol Med. 2019;11. 10.15252/emmm.201809992 PMC6404110 30770338

[29] Eder C, Wild C (2019). Technology forecast: advanced therapies in late clinical research, EMA approval or clinical application via hospital exemption. J Mark Access Health Policy.

[30] Advanced therapies with CAR-T approach plan2019. https://www.mscbs.gob.es/profesionales/farmacia/Terapias_Avanzadas.htm. Accessed May 2021.

[31] BIFIMED: Buscador de la Información sobre la situación de financiación de los medicamentos—Nomenclátor de ABRIL—2021: NC1. https://www.mscbs.gob.es/profesionales/medicamentos.do?metodo=buscarMedicamentos. Accessed May 2021.

